# Using eDNA to understand predator–prey interactions influenced by invasive species

**DOI:** 10.1007/s00442-023-05434-6

**Published:** 2023-08-18

**Authors:** Maria Riaz, Dan Warren, Claudia Wittwer, Berardino Cocchiararo, Inga Hundertmark, Tobias Erik Reiners, Sven Klimpel, Markus Pfenninger, Imran Khaliq, Carsten Nowak

**Affiliations:** 1 Conservation Genetics Section, Senckenberg Research Institute and Natural History Museum, 63571 Frankfurt, Gelnhausen Germany; 2 LOEWE Centre for Translational Biodiversity Genomics (LOEWE-TBG), Senckenberganlage 25, 60325 Frankfurt Am Main, Germany; 3 Faculty of Biological Sciences, Institute for Ecology, Evolution and Diversity, Goethe University, Max-Von-Laue-Straße 9, 60438 Frankfurt Am Main, Germany; 4 Biodiversity and Biocomplexity Unit, Okinawa Institute of Science and Technology Graduate University, Okinawa, Japan; 5 Senckenberg Biodiversity and Climate Research Centre (BiK-F), Senckenberganlage 25, 60325 Frankfurt Am Main, Germany; 6 Hessische Gesellschaft Für Ornithologie Und Naturschutz (HGON E. V.), Lindenstrasse 5, 61209 Echzell, Germany; 7 Institute for Molecular and Organismic Evolution, Johannes Gutenberg University, Johann-Joachim-Becher-Weg 7, 55128 Mainz, Germany; 8 Department of Education, Punjab, Pakistan; 9 Department of Aquatic Ecology Eawag (Swiss Federal Institute of Aquatic Science and Technology) Überlandstrasse 133, 8600 Dübendorf, Switzerland; 10 Snow and Landscape Research (WSL), Swiss Federal Institute for Forest, Flüelastr. 11, 7260 Davos Dorf, Switzerland

**Keywords:** Amphibian decline, Biotic interactions, Environmental DNA, Invasive species, Predator–prey interactions

## Abstract

Invasive predatory species may alter population dynamic processes of their prey and impact biological communities and ecosystem processes. Revealing biotic interactions, however, including the relationship between predator and prey, is a difficult task, in particular for species that are hard to monitor. Here, we present a case study that documents the utility of environmental DNA analysis (eDNA) to assess predator–prey interactions between two invasive fishes (*Lepomis gibbosus*, *Pseudorasbora parva*) and two potential amphibian prey species, (*Triturus cristatus*, *Pelobates fuscus*). We used species-specific TaqMan assays for quantitative assessment of eDNA concentrations from water samples collected from 89 sites across 31 ponds during three consecutive months from a local amphibian hotspot in Germany. We found a negative relationship between eDNA concentrations of the predators (fishes) and prey (amphibians) using Monte-Carlo tests. Our study highlights the potential of eDNA application to reveal predator–prey interactions and confirms the hypothesis that the observed local declines of amphibian species may be at least partly caused by recently introduced invasive fishes. Our findings have important consequences for local conservation management and highlight the usefulness of eDNA approaches to assess ecological interactions and guide targeted conservation action.

**Supplementary Information:**

The online version contains supplementary material available at 10.1007/s00442-023-05434-6.

## Introduction

Revealing biotic interactions is a challenging but important task to understand the ecological integrity and functioning of biological communities (Beauchamp et al. 2007; Lee et al. 2019). The intrinsic complexity of biotic interactions poses a particular difficulty when aiming to understand predator–prey interactions in aquatic habitats (Campanella et al. 2019). This challenge can be partly attributed to established methods for monitoring the presence and abundance of species, including direct catch (Haubrock et al. 2020), electrofishing (Allard et al. 2014), radio telemetry, hydroacoustics (Campanella et al. 2019), visual counting, and trawls (Rodgers et al. 2017; Stevenson 2018). These methods are highly dependent upon the probability of species being present at a specific time and place, the effects of water quality on the visual census, and the investigator’s expertise and level of sampling effort (Jerde et al. 2011; Hayward et al. 2015). In addition, some of the above-listed tools are somewhat invasive and therefore detrimental to the monitored species and may disturb the habitat to various degrees (Meyer et al. 2021).

In recent times, DNA-based studies have demonstrated promising and novel insights for evaluating predator–prey interactions in terrestrial and aquatic habitats (Roslin and Majaneva 2016). For instance, gut contents have been used to reveal trophic interactions, population structure and feeding preferences in pioneer sites of glacier forelands (Sint et al. 2019), predatory vampire bats (Bohmann et al. 2018), fisheries discard in marine fauna (Lejeune et al. 2022), terrestrial arthropods (Paula et al. 2016), spiders (Saqib et al. 2021) and among coral reefs (Casey et al. 2019). However, the application and resolution of invasive genetic methods involving catching and sampling of organisms may be unsuitable for rare, endangered, or elusive species. A robust, sensitive, and widely applicable non-invasive monitoring method to assess species interactions would therefore be of considerable importance given the rapid spread of invasive species in the Anthropocene (Cucherousset and Olden 2011).

Environmental DNA (eDNA) as a non-invasive and robust assessment method has undergone rapid improvement during the past decade, involving quantitative detection of single species as well as metabarcoding-based assessment of entire communities (Taberlet et al. 2012; Thomsen et al. 2012; Bálint et al. 2017). Interestingly, however, only a few studies to date have assessed the potential of eDNA beyond mere species detection (Yamanaka and Minamoto 2016; Pawlowski et al. 2018; Riaz et al. 2020), pathogen surveillance (Mosher et al. 2017), and diet analysis to reveal trophic network structures (Thomsen and Sigsgaard 2019; Djurhuus et al. 2020; Meyer et al. 2020; D’Alessandro and Mariani 2021; Banerjee et al. 2022). eDNA may give a boost to the fields of ecology and population dynamics, particularly because of its ability to detect rare, unseen individuals for nearly all taxon types (Ficetola et al. 2008; Herder et al. 2014; Keskin 2014; Hunter et al. 2015) and across different habitats (Bohmann et al. 2014; Thomsen and Willerslev 2015; Sasso et al. 2017).

Here, we explored the potential of eDNA for assessment of biotic interactions using an aquatic study system involving invasive predatory fishes and locally endangered amphibian species as potential prey. In aquatic ecosystems, biological invasions of predatory fish species may lead to increased competition and can result in the restructuring of trophic interactions (Bishop et al. 2012) which influence prey species abundances (Allentoft and O’Brien 2010). For instance, predation by invasive fish species is one critical factor in the decline of amphibians worldwide (Davidson and Knapp 2007; Préau et al. 2017; Morisette et al. 2021). Since the 1980s, amphibians have declined globally at an alarming rate (Sodhi et al. 2008), which is currently about 200 times higher than the historical rate (Collins 2010). While complex interactions of several anthropogenic factors (Knapp and Matthews 2000; Miró et al. 2018) are responsible for these declines, the extended aquatic larval periods of many amphibian species make them highly vulnerable to predation by fishes. Predation thus has substantial impact on the diversity and distribution of amphibian communities due to restricting their ranges, leading to rapid population declines and ultimately extirpation of many species (Kats and Ferrer 2003; Hartel et al. 2007).

We tested the impact of the presence of two predatory fish species that recently invaded a protected wetland area in Germany on two native amphibian species using species-specific eDNA detection from water samples. The main objectives of this study were to test if (a) eDNA-based detection can be used as a fast and minimal invasive tool to reveal predator–prey-interactions in aquatic environments, and (b) the observed ongoing decline of both amphibian species may be correlated with the recent invasions of predatory fishes.

## Materials and methods

### Study area and species

**Fig. 1:**
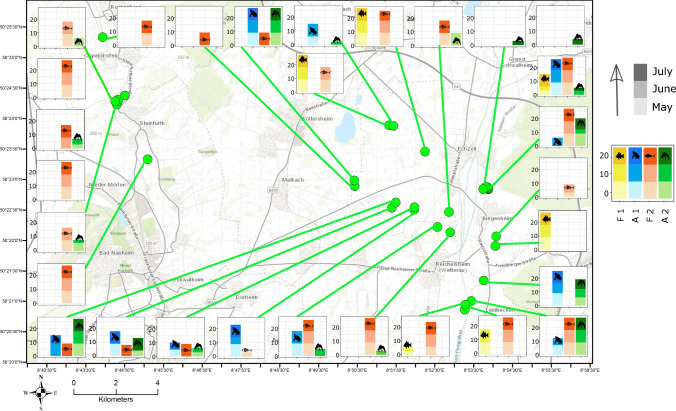
Map of the study area with sampling locations and eDNA detections at each site during the three sampling months. Green points represent the sampling sites. F1 and F2 represent *Lepomis gibbosus* and *Pseudorasbora parva* and A1 and A2 indicate *Pelobates fuscus* and *Triturus cristatus*. The bar charts around the study sites show the detections across three months at each site. Blue and green colors denote prey species and red and yellow colors show predator species. Color gradient indicates months in ascending order (Bottom month of May, middle June and top July). The arrangement of species is as given in the legend for each bar

As study area, we chose a part of the “Auenverbund Wetterau”, located approximately 30 km north of Frankfurt am Main in the federal state of Hessen, Germany (Fig. 1). The sampling sites include seven locally important nature protection areas of approx. 70 km^2^ that provide breeding habitats for several protected species of birds and amphibians and aim at protecting species-rich aquatic and wetland communities. The “Auenverbund Wetterau” is included as a landscape protection area within the European Natura 2000 network of protected areas (AGAR and Fena 2010). It consists of several ponds of varying sizes as well as meadows and reeds that are regularly flooded in spring and autumn, providing favorable habitats for numerous wetland birds, amphibians, and other taxa. Ten out of twenty-one amphibian species native to Germany are found in the area (Franke 2013; Geske and Stübing 2014).

The potential prey species selected for this study are two amphibian species, the garlic toad, *Pelobates fuscus* (hereafter A1), and the crested newt, *Triturus cristatus* (A2). Globally, both species are of least concern, but with decreasing population trends (IUCN/SSC 2009). Locally, A1 is listed as critically endangered in the Red List of the Federal State of Hessen (AGAR and Fena 2010). A2 is also a protected species under European and UK legislation and is listed in the EU Habitats Directive’s Appendix II and IV (Edgar and Bird 2006a). Hence, Natura 2000 has designated several protected areas for both species. We further selected two predatory fish species, *Lepomis gibbosus* (F1) and *Pseudorasbora parva* (F2), which are successful invaders (Garcia-Berthou and Moreno-Amich 2000) and listed as invasive species of particular concern by the European Union (European Environment Agency 2012).

### Traditional survey

To confirm target species presence, we conducted a traditional survey in parallel with the eDNA survey for both predator and prey species by established methods including bioacoustic monitoring (for A1) as well as placing amphibian and fish traps (for A2, F1, F2) in several of the studied ponds. To catch crested newts (A2) as well as occasionally the other targeted species (A1, F1, F2), we used a combination of commonly used bucket traps (following Ortmann 2009) and bottle traps to cover different areas within the ponds. Bottle traps were placed in the open water zone close to the shore and bucket traps were floated in the water to catch emerging newts or amphibian larvae. Traps were placed in different ponds from April (when only adults are present) through July (to catch larvae). These traps were not baited to avoid the attraction of predators such as fishes, aquatic insects, and leeches. However, both of the traps may not be efficient for catching frogs and toads, so we performed acoustic surveys for garlic toads (A1) to ensure the presence/absence of the species (Table 1, Supplementary material).

We used fish bait traps from Behr fishing with a size of 55 cm, a mesh size of 3–4 mm, and two openings of 6 cm size to catch F1 and F2. Fish traps were not completely sunk into the water due to the risk of bycatch of amphibians and small mammals. The traps were baited with Frolic dog food and placed for a maximum of one night per catch to prevent animals from dying. We avoided the use of scoop nets to prevent vegetation damage in these nature reserves.

### eDNA sampling

In this study, we sampled 31 ponds during the breeding season of amphibians for three consecutive months from May 2018 to July 2018 (Fig. 1; Table 2, Supplementary material). We sampled all ponds during the first week of each sampling month in a consistent pattern to create a uniform sampling intervals. We took three samples from each site in each month, summarizing a total of nine samples collected from each pond during the study period. Some sites were not sampled during June or July due to the presence of sensitive migratory birds or the drying of the pond (Table 2, Supplementary material). In addition to this, We also took 1 L surface water samples from ponds with a known absence of all study species as negative field controls.

We collected water samples by the grab-and-collect method following Riaz et al. (2020) from pond surface, middle, and above the substrate depending upon the pond depth. Specimens were pooled to obtain a representative sample of the water body being studied and filtered on the same day through sterile 0.45 μm Sterivex-GP filters (Millipore Merck, KGaA, Germany) using 50 ml syringes (Omnifix™ Solo 3-piece, Fisher Scientific) following Sigsgaard et al. (2016). The volume of filtered water ranged between 100 and 600 ml, depending upon the clogging of the filter as a result of turbidity of the sampled pond.

DNA filtration took place in a designated eDNA laboratory, adhering to rigorous clean laboratory practices. This involved sterilizing the working benches using a 10% bleach solution and ensuring frequent glove changes for maximum cleanliness and contamination prevention. Sterivex filters were stored at  – 20 °C until subsequent DNA extraction. We also took 1L surface water samples from ponds with a known absence of all study species and filtered them as negative field controls. Additionally, for each sampling event, equipment and field blanks were taken to monitor the risk of contamination from the equipment or materials used during the process as recommended in other studies (Piaggio et al. 2014; Riaz et al. 2020).

For assay validation, we included water samples from aquaria as positive controls of qPCR for all species except A2. In total, 310 water samples including 267 field samples, negative controls, and field and equipment blanks were collected in the course of this study (Table 2, Supplementary material). Before each sampling event, we decontaminated all equipment using 0.25% peracetic acid or 10% bleach solution, rinsing with 96% ethanol, and then exposed to UV light for a minimum of 40 min (Riaz et al. 2018).

### Primer design and testing

We designed and tested assays for predatory fish species (F1 and F2) by following the validation scale provided by DNAquaNet (Thalinger et al. 2021). We extracted DNA from tissue samples of the four study species (N = 20) as well as several co-occurring amphibian and fish species (Table 3, Supplementary material) using the QIAGEN Blood and Tissue Kit (QIAGEN GmbH, Germany) as recommended by the manufacturer’s instructions. DNA extracts were subsequently quantified with NanoDrop™ Spectrophotometer (Thermo Fisher Scientific, Waltham, MA, USA). We designed a TaqMan® MGB assay for F1 (*L. gibbosus*) (forward primer 5′-TCCACATCGGTCGAGGACTA-3′; reverse primer 5′-CGACTCCGATGTTTCATGTTTC-3′; TaqMan® MGB probe 5' Dye: 6FAM-ATTATGGCTCTTACCTTTAC-MGBNFQ) and another TaqMan® MGB assay for F2 (*P. parva)* (forward primer 5′-AACAGGACTATTCTTGGCCATACAC-3′; reverse primer 5′-GATGTGGGCCACCGATGA-3′; TaqMan® MGB probe 5' Dye: 6FAM-TCTGACATCTCAACTGCA-MGBNFQ) on cytb gene region by using Primer Express 3.0.1 (Life Technologies). For testing assay specificity and sensitivity, we performed the multistep process for assay validation including in silico testing, in vitro testing, and in situ testing following Thalinger et al. (2021). For in silico testing, we performed primer BLAST searches (Ye et al. 2012) with all promising primer pair combinations. We selected the primer pairs that fully matched the target species’ reference sequences while including several mismatches for the non-target fish species. For in vitro testing, we tested both assays in wet laboratory conditions for specificity by analyzing DNA extracts of each target species, a set of 36 local fish and lamprey, 11 amphibian species (Table 3, Supplementary material), and human DNA by qPCR, following thermal conditions of Riaz et al. (2018) at 50 °C for 5 min, 95 °C for 10:20 min followed by 50 cycles of 95 °C for 15 s and 62 °C as annealing temperature for 30 s. For validating assay sensitivity, we set up a standard curve with an 8-level dilution series starting with a DNA concentration of 50 ng. Each dilution level was replicated 20 times per sample to conduct a SIMQUANT analysis for assay validation (Berdal et al. 2008). PCR efficiency, the limit of quantification (LOQ), and the limit of detection (LOD) (Table 4, Supplementary material) were also determined using the formula (y = ( –  1.526) 9 ln(x) + 41.232) following Riaz et al. (2018). For in situ testing, the assay was tested on (i) water samples from aquaria with each target species and (ii) water samples from a pond with known absence of all target species to address the possibility of producing false negative and false positive results. Finally, we Sanger sequenced tissue samples of both fish species and eDNA samples from four positive and four negative study sites (Table 5, Supplementary material) using M13 tagged species-specific assays. We used the same qPCR thermal conditions as mentioned above, purified the PCR product by adding 2 μl ExoSAP-IT™ containing 4 units/μl Exonuclease I and 1.6 units|μl FastAP (Thermofisher Scientific) and incubated at 37 °C for 15 min after a denaturation step at 80 °C for 15 min. The purified PCR products were diluted (3 × 48 μl PCR water:1 μl PCR product) and sequenced on a ABI 3730xl Sanger sequencer (Thermo Fisher). We selected sites for sequencing based on the information obtained through traditional monitoring results and qPCR detection/non-detection. We sequenced two replicates from each site including sites with detection of both, one, and no targeted species. We analyzed the sequences using Sequence Scanner Software v1.0 (Applied Biosystems**®**). The sequence information is submitted to GenBank and accession numbers are provided (Table 5, Supplementary material).

### Analysis of eDNA samples

We extracted DNA from all water samples (*N* = 267) using the Qiagen Blood and Tissue Kit (Qiagen GmbH, Germany) following Sigsgaard et al. (2016) and running the Fast DNA Stool Kit (Qiagen) protocol on the QIAcube automated DNA extraction system (Qiagen) as recommended by manufacturer’s instructions. All extractions were performed under UV hoods with positive air pressure in a laboratory dedicated to the pre-PCR processing of environmental samples with low DNA content (Taberlet et al. 1999).

For the amphibians A1 and A2, we used species-specific TaqMan® MGB qPCR assays from Thomsen et al. (2012) and established an 8-level dilution series with a starting DNA concentration of 50 ng. Further steps for determining PCR efficiency, LOQ, and LOD were performed following assays of predator species (Table 4, Supplementary material). We performed qPCR reactions on QuantStudio® 3 (Thermo Fisher Scientific) using TaqMan® Environmental Master Mixture 2.0 (Lifetechnologies, part of Thermo Fisher Scientific, USA) following the thermal conditions described in Riaz et al. (2018) as shown above. All qPCR runs included field samples in triplicates, six levels of standard dilution series as the positive control, a no template control (PCR water), and either an equipment/field control or an extraction blank in duplicates to ensure the reliability of the results.

We analyzed the qPCR runs with Quantstudio® software v1.1 and calculated relative DNA amounts based on the standard curve, and obtained Cq values (Table 4, Supplementary material).

A site was scored as positive if there was an amplification of at least two biological (different water samples from one site) or two technical (PCR replicate of the same sample) replicates from the same site and same month with DNA quantity above the detection threshold (LOD) for each target species (Riaz et al. 2018).

### Inhibition test

We used TaqMan® Environmental Master Mix 2.0 to minimize potential inhibition effects (Jane et al. 2015). To test for inhibition, we randomly selected and tested a set of five dark-colored and five light-colored sample extracts, diluted them into 5, 10, and 100 folds (Riaz et al. 2020), and run qPCR reactions in triplicates.

### Statistical analysis

**Fig. 2:**
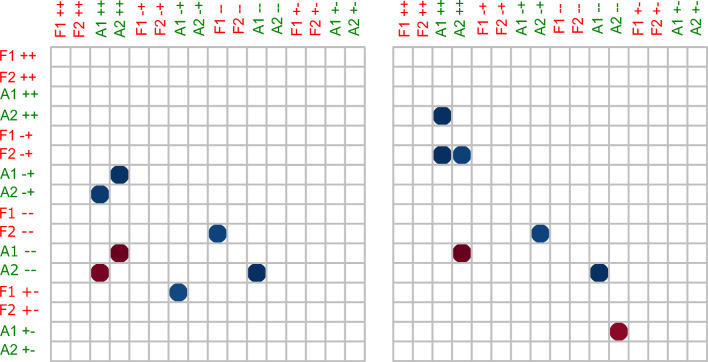
Results of Monte-Carlo Tests for the months of May–June (right) and June-July (left) showing the negative correlation between two predators and two prey species. *Lepomis gibbosus* represents as F1, *Pseudorasbora parva* as F2, *Pelobates fuscus* as A1 and *Triturus cristatus* as A2. Events are represented as “persist with +  + ”, “enter with—+ ”, “stay zero with –” and “disappear with + -”. The blue dot color indicates that an event happened more than expected by chance and red dot color represents when an event happened less than expected by chance

We followed a two-step analytical approach to account for site effects and temporal autocorrelation. The first step was a Monte Carlo test in which we used eDNA to estimate species presence/absence based on eDNA detection/non-detection at each site for a pair of consecutive months (May–June or June–July) and to find out whether transitions between states for one species were correlated with transitions for other species. We considered four types of transitions for each species between subsequent months: (i) a species could be absent in the first month and remain absent in the following month (which we termed “stay zero as –”), (ii) a species could be present in both months (“persist as +  + ”), (iii) a species could be absent in the first month and present in the next month (“enter as—+ ”) (iv) or could be present in the first month and absent in the second month (“disappear as + –”). We constructed a matrix measuring the number of times where the event on the horizontal was accompanied by the events on the vertical, divided by the number of times the event on the horizontal happened (Fig. 2). In other words, if half of the times when species A persisted in a population were accompanied by species B being absent, the value for the row representing “species A persists” and the column “species B disappears” would be 0.5. If species B only disappeared when species A persisted, however, the accompanying proportion above the diagonal would be 1.

From this, we implemented a Monte-Carlo test to establish whether transitions in prey states were correlated with the presence of predators. In this test, we permuted the transitions across sites for each species separately and recorded how often transition types for different species co-occurred. Repeating this process many times allowed us to construct a null distribution of expected co-occurrence of transitions between species that accounted for autocorrelation in states between consecutive months as well as the overall frequency of each transition type for each species. By contrasting our empirical observations with these null distributions, we assessed the statistical support for the hypothesis that certain transition types co-occurred more often than expected by chance. Data analysis was performed in R version 4. 0. 2 (R Core Team 2020), R code and data were provided as public-for-peer review via the following github link: https://github.com/Maria-289* Imran/eDNA_biotic_interaction_MS.* and in supporting material (Table 6, Supplementary material).

## Results

213 of 267 (80%) analyzed samples from 89 sites (Fig. 1; Table 2, Supplementary material) collected from May to July 2018 showed positive detections of at least one target species. The presence of at least one of the four target species occurred in 76 sites (85%); amphibian prey species at 45% (21% with amphibians only), while predatory fish were found at 63% (38% with fish only). Predator and potential prey species co-occurred at 25% of the sites when ignoring different sampling months. eDNA detected all target species at more sites compared to the traditional survey except for A1, which was detected at four additional sites in the acoustic survey (see Table 1, Supplementary material).

Results from Monte Carlo tests showed that the two amphibian prey species co-existed more often than expected by chance, while the two predator species tend to be absent from the same sites more often than expected. The transitions indicated between May–June showed that the entry of A1 in a pond was strongly correlated with an absence of F2, suggesting a predation effect of the predatory fish species. Transitions between June-July also showed a significant negative relationship between F2 and both amphibian prey species, A1 and A2. Most sites were successfully occupied by the two prey species only when the predator was absent. We also found that F2 invaded the ponds when either one or both of the prey species persisted. Moreover, A1 co-occurred with A2 more often than expected by chance as shown by the transitions obtained during the entire study period.

## Discussion

Assessing biotic interactions is an important topic both in fundamental and applied ecology, and improves our understanding of population dynamics and community assemblages (Wisz et al. 2013; Fraser et al. 2020). Here, our case study aims to demonstrate the feasibility of eDNA techniques to investigate predator–prey interactions in freshwater ecosystems. While all ponds were historically inhabited by amphibians, including our target species (Geske and Stübing 2014), we found that both amphibian prey species showed complete absence or at least underwent a sharp decline in those ponds occupied by invasive predatory fishes during the study period.

Interestingly, we found that both prey species (A1, A2) showed a negative relationship with fish predators and were inclined to be drawn towards the predator-free ponds from which predator species had disappeared or had not yet occupied, to complete their breeding process. Another study found that higher abundances of amphibian larvae and adults were recorded in a fish-free protected area compared to an adjacent protected area that had fish, suggesting the potential effect of predation and avoidance of prey species to predator presence (Knapp and Matthews 2000). A congruent negative relationship has also been observed in other studies (e.g. Sodhi et al. 2008; Haubrock and Altrichter 2016; Miró et al. 2020; Kačergytė et al. 2021). Meanwhile, we found that the predator species entered ponds where one or both prey species persisted, providing additional evidence for a relationship between predator and prey. Our results are also supported by the traditional monitoring data that found a rapid spread of the predator species over the year into the ponds inhabited by prey species (Geske and Stübing 2014; Stübing and Hundertmark 2015).

We are aware of the fact that several factors, such as seasonal changes, environmental variables and diseases significantly affect the presence and interactions of species (Davis et al. 2017). In our study system, the relationship between predators and prey appeared strongest during the amphibian breeding season, when predators mainly feed on the larvae. Predation pressure on amphibians decreases once amphibians reach maturity and ultimately leave the water body. We consequently studied the predator–prey relationship only during the amphibians’ breeding season when the probability of interaction was highest.

Besides the obvious seasonal effects, other environmental variables may potentially influence our interpretation by causing a decline in the amphibian population. Warmer and drier conditions, for instance, may be challenging for amphibians because of dehydration and fluctuations in body temperatures. Warmer climatic conditions may hamper amphibians daytime foraging activity which can influence fitness in prey species. Similarly, the limitations in movement under warmer and drier conditions may hamper amphibian’s ability to find suitable microhabitats (Pounds et al. 2006). Here, our study period was too short (restricted to only a few months) to discern any strong effect by environmental variables as observed in other co-relational studies such as the well-known sardine-anchovy-relationship where environmental variables have been found to have a significant impact on the population dynamics of these fish species (Sugihara et al. 2012). Warmer conditions may also increase the prevalence of some diseases (Anchukaitis and Evans 2010; Rohr and Raffel 2010; Bartelt et al. 2022) by providing optimum temperatures for pathogen species such as the fungus *Batrachochytrium dendrobatidis* (BD) (Geske and Stübing 2014). However, ponds in our study area were frequently monitored as part of the protected areas where BD has not been reported.

Differences in habitat preferences of both prey and predator species found in our study may be attributed to pond size and pond accessibility. Pond size likely did not affect our results since both predator and prey species were detected in ponds of all size categories (Table 1, Supplementary material). Traditional monitoring data of fish and amphibians based upon different sampling techniques including trapping, visual observations, and call records also detected both groups of species in small as well as large ponds (Stübing and Hundertmark 2015), supporting the assumption of negligible effect of pond size (e.g., see Kačergytė et al. 2021). Moreover, the majority of study ponds (both predator-abundant and prey-abundant ponds) were located merely meters apart without any physical barrier. As such, pond inaccessibility due to geographical barriers may have little impact on the settlement of prey species in ponds that have dense populations of predator species. In general, many amphibian species tend to return to the same breeding sites each year including *T. cristatus* and *P. fuscus* (Edgar and Bird 2006), showing the breeding site fidelity. However, the traditional collection data showed the gradual population shrinkage/shift of the prey species in several of the studied breeding ponds (Stübing and Hundertmark 2015). Thus, it appears likely that the reduction or absence of amphibian species in these study ponds might indeed be due to the spread of predatory fish species.

The observed evidence for the detrimental effects of invasive predatory fishes on amphibians in our study is supported by recent data from Haubrock et al. (2019) who found the presence of larvae of the garlic toad and crested newt in the stomach contents of *P. parva* from our study area. These authors addressed trophic interactions and potentially detrimental effects of the invasive predatory fish species on several native prey species, including amphibians.

Several potential pitfalls may have impact our study, given the correlational nature of our findings of predator–prey interactions. First, we acknowledge that eDNA can only provide partial quantitative information concerning the number of individuals, their relative abundance and biomass of the target species compared to traditional survey methods (Beng and Corlett 2020; Spear et al. 2020; Yates et al. 2019). However, the invasiveness of traditional survey methods may not be suitable for these protected areas that serve as the breeding habitats of many rare and protected species of birds and amphibians. Second, amphibians are semi-aquatic organisms who leave their aquatic habitats after the breeding season. The sharp declines in prey numbers may thus be impacted by the fact that in the late sampling phase most amphibians may have already left the water, leading to reduced detectability with eDNA. However, the traditional monitoring surveys carried out in parallel with our last eDNA sampling period (early July) detected the presence of the prey species (both A1 and A2) in most of these studied ponds (Appendix S1, Supporting material). Similarly, previous traditional monitoring data collected between 2015 and 2020 in the same study area confirmed the presence of amphibians in the sampling months of May–July (Geske and Stübing 2014; Stübing and Hundertmark 2015) and also documented the gradual disappearance of the amphibian prey species and parallel spread of predatory fishes in many of these ponds, supporting the assumption of influence of predator on prey species. Third, the testing of eDNA persistence is important to inform whether the detected sequence derived from pre-existing DNA traces or from animals that were present at the sampling sites during sample collection (Dejean et al. 2011; Harrison et al. 2019). While, we cannot exclude the possibility of some impact here, the comparison of traditional survey results in parallel with the eDNA survey to ensure the joint presence of predator and prey species in the study sites (Appendix S1, Supporting material) allows us to refute severe bias here.

Our findings provide empirical evidence for the applicability of eDNA techniques to study predator–prey interactions with limited effort and low disturbance in the field. The detection of a significant environmental signal of biotic interactions in the short time period of this study provided strong evidence for the utility of eDNA in studying predator–prey interactions. We argue that eDNA detection can also be applied to reveal other biotic interactions important to biodiversity. While we chose a rather simple system with only four species, we believe that eDNA-based species detection may be used in larger-scale analyses of biotic interactions within complex communities, as possible with metabarcoding (Wilcox et al. 2018; Djurhuus et al. 2020; Kačergytė et al. 2021) or specific platforms allowing for multispecies detection, such as Fluidigm (Wilcox et al. 2020; Riaz et al. unpublished). Our example demonstrates that eDNA analysis can provide important insights into possible causes of population decline, and we therefore recommend the inclusion of this tool in conservation programs to elucidate the causes of regional biodiversity loss.

## Supplementary Information

Below is the link to the electronic supplementary material.

Supplementary file1 (DOCX 55 KB)

## Data Availability

Data can be found in electronic supplementary material.

## Declarations

### Code availability

Data and code are provided as public-for-peer review via the following github link: https://github.com/Maria-Imran/eDNA_biotic_interaction_MS.

## References

[CR1] AGAR, Fena (2010) Rote Liste der Reptilien und Amphibien Hessens (Reptilia et Amphibia)

[CR2] Allard L, Grenouillet G, Khazraie K (2014). Electrofishing efficiency in low conductivity neotropical streams: towards a non-destructive fish sampling method. Fish Manag Ecol.

[CR3] Allentoft ME, O’Brien J (2010). Global amphibian declines, loss of genetic diversity and fitness: a review. Diversity.

[CR4] Anchukaitis KJ, Evans MN (2010). Tropical cloud forest climate variability and the demise of the Monteverde golden toad. Proc Natl Acad Sci U S A.

[CR5] Bálint M, Nowak C, Márton O, et al (2017) Twenty-five species of frogs in a liter of water: eDNA survey for exploring tropical frog diversity. 0–36. 10.1101/176065. 10.1111/1755-0998.12934

[CR6] Banerjee P, Stewart KA, Antognazza CM (2022). Plant–animal interactions in the era of environmental DNA (eDNA)—A review. Environ DNA.

[CR7] Bartelt PE, Thornton PE, Klaver RW (2022) Modelling physiological costs to assess impacts of climate change on amphibians in Yellowstone National Park, U.S.A. Ecol Indic 135:108575. 10.1016/j.ecolind.2022.108575

[CR8] Beauchamp DA, Wahl DH, Johnson BM (2007) Predator-Prey Interactions. American Fisheries Society

[CR9] Beng KC, Corlett RT (2020) Applications of environmental DNA (eDNA) in ecology and conservation: opportunities, challenges and prospects. Springer Netherlands. 10.1007/s10531-020-01980-0

[CR10] Berdal KG, Bøydler C, Tengs T, Holst-Jensen A (2008). A statistical approach for evaluation of PCR results to improve the practical limit of quantification (LOQ) of GMO analyses (SIMQUANT). Eur Food Res Technol.

[CR11] Bishop PJ, Mainguy G, Angulo A (2012). The amphibian extinction crisis - what will it take to put the action into the Amphibian conservation action plan?. SaPIEnS.

[CR12] Bohmann K, Evans A, Gilbert MTP (2014). Environmental DNA for wildlife biology and biodiversity monitoring. Trends Ecol Evol.

[CR13] Bohmann K, Gopalakrishnan S, Nielsen M (2018). Using DNA metabarcoding for simultaneous inference of common vampire bat diet and population structure. Mol Ecol Resour.

[CR14] Campanella F, Auster PJ, Christopher Taylor J, Muñoz RC (2019). Dynamics of predator-prey habitat use and behavioral interactions over diel periods at sub-tropical reefs. PLoS ONE.

[CR15] Casey JM, Meyer CP, Morat F (2019). Reconstructing hyperdiverse food webs: Gut content metabarcoding as a tool to disentangle trophic interactions on coral reefs. Methods Ecol Evol.

[CR16] Collins JP (2010). Amphibian decline and extinction: what we know and what we need to learn. Dis Aquat Organ.

[CR17] Cucherousset J, Olden JD (2011). Feature : introduced fish and ecology ecological impacts of non-native freshwater fishes. Fisheries.

[CR18] D’Alessandro S, Mariani S (2021). Sifting environmental DNA metabarcoding data sets for rapid reconstruction of marine food webs. Fish Fish.

[CR19] Davidson C, Knapp RA (2007). Multiple stressors and amphibian declines: Dual impacts of pesticides and fish on yellow-legged frogs. Ecol Appl.

[CR20] Davis CL, Miller DAW, Walls SC (2017). Species interactions and the effects of climate variability on a wetland amphibian metacommunity. Ecol Appl.

[CR21] Dejean T, Valentini A, Duparc A (2011). Persistence of environmental DNA in freshwater ecosystems. PLoS ONE.

[CR22] Djurhuus A, Closek CJ, Kelly RP (2020). Environmental DNA reveals seasonal shifts and potential interactions in a marine community. Nat Commun.

[CR23] Edgar P, Bird DR (2006) Action Plan for the conservation of the crested newt Triturus cristatus species complex in Europe - Convention on the Conservation of European Wildlife and Natural Habitats. Standing Comittee, 26th meeting, Strasbourg, 27–30 November 2006.

[CR24] European Environment Agency (2012) European waters — assessment of status and pressures. European Environment Agency

[CR25] Ficetola GF, Miaud C, Pompanon F, Taberlet P (2008). Species detection using environmental DNA from water samples. Biol Lett.

[CR26] Franke NM (2013) Die Geschichte des Naturschutzes in Hessen. Hessisches Ministerium für Umwelt, Energie, Landwirtschaft und Verbraucherschutz

[CR27] Fraser D, Soul LC, Tóth AB (2020). Investigating biotic interactions in deep time. Trends Ecol Evol.

[CR28] Garcia-Berthou E, Moreno-Amich R (2000). Introduction of exotic fish into a Mediterranean lake over a 90-year period. Arch Fur Hydrobiol.

[CR29] Geske C, Stübing S (2014). Vergleichende Untersuchungen zur Bestandsgröße eines Vorkommens der Knoblauchkröte (Pelobates fuscus) im Bingenheimer Ried in der Wetterau (Hessen). Zeitschrift Fur Feldherpetologie.

[CR30] Harrison JB, Sunday JM, Rogers SM (2019). Predicting the fate of eDNA in the environment and implications for studying biodiversity. Proc R Soc B Biol Sci.

[CR31] Hartel T, Nemes S, Cogǎlniceanu D (2007). The effect of fish and aquatic habitat complexity on amphibians. Hydrobiologia.

[CR32] Haubrock PJ, Altrichter J (2016). Northern crested newt (Triturus cristatus) migration in a nature reserve: Multiple incidents of breeding season displacements exceeding 1km. Herpetol Bull.

[CR33] Haubrock PJ, Balzani P, Azzini M (2019). Shared histories of co-evolution may affect trophic interactions in a freshwater community dominated by alien species. Front Ecol Evol.

[CR34] Haubrock PJ, Azzini M, Balzani P (2020). When alien catfish meet—Resource overlap between the North American Ictalurus punctatus and immature European Silurus glanis in the Arno River (Italy). Ecol Freshw Fish.

[CR35] Hayward MW, Boitani L, Burrows ND (2015). Ecologists need robust survey designs, sampling and analytical methods. J Appl Ecol.

[CR36] Herder JE, Valentini A, Bellemain E, et al (2014) Environmental DNA - a review of the possible applications for the detection of (invasive) species. Sticht RAVON, Nijmegan Rep 10.13140/RG.2.1.4002.1208

[CR37] Hunter ME, Oyler-McCance SJ, Dorazio RM (2015). Environmental DNA (eDNA) sampling improves occurrence and detection estimates of invasive Burmese pythons. PLoS ONE.

[CR38] IUCN/SSC (2009) Pelobates fuscus. The IUCN Red List of Threatened Species 2009

[CR39] Jane SF, Wilcox TM, Mckelvey KS (2015). Distance, flow and PCR inhibition: EDNA dynamics in two headwater streams. Mol Ecol Resour.

[CR40] Jerde CL, Mahon AR, Chadderton WL, Lodge DM (2011). “Sight-unseen” detection of rare aquatic species using environmental DNA. Conserv Lett.

[CR41] Kačergytė I, Petersson E, Arlt D (2021). Environmental DNA metabarcoding elucidates patterns of fish colonisation and co-occurrences with amphibians in temperate wetlands created for biodiversity. Freshw Biol.

[CR42] Kats LB, Ferrer RP (2003). Alien predators and amphibian declines: Review of two decades of science and the transition to conservation. Divers Distrib.

[CR43] Keskin E (2014). Detection of invasive freshwater fish species using environmental DNA survey. Biochem Syst Ecol.

[CR44] Knapp RA, Matthews KR (2000). Non-native mountain fish introductions and the decline of the mountain yellow-legged frog from within protects areas. Conserv Biol.

[CR45] Lee CK, Laughlin DC, Bottos EM (2019). Biotic interactions are an unexpected yet critical control on the complexity of an abiotically driven polar ecosystem. Commun Biol.

[CR46] Lejeune B, Mouchet MA, Mehault S, Kopp D (2022). Gut content metabarcoding reveals potential importance of fisheries discards consumption in marine fauna. Can J Fish Aquat Sci.

[CR47] Meyer JM, Leempoel K, Losapio G, Hadly EA (2020). Molecular ecological network analyses: an effective conservation tool for the assessment of biodiversity, trophic interactions, and community structure. Front Ecol Evol.

[CR48] Meyer A, Boyer F, Valentini A (2021). Morphological vs. DNA metabarcoding approaches for the evaluation of stream ecological status with benthic invertebrates: Testing different combinations of markers and strategies of data filtering. Mol Ecol.

[CR49] Miró A, Sabás I, Ventura M (2018). Large negative effect of non-native trout and minnows on Pyrenean lake amphibians. Biol Conserv.

[CR50] Miró A, O’Brien D, Tomàs J (2020). Rapid amphibian community recovery following removal of non-native fish from high mountain lakes. Biol Conserv.

[CR51] Morisette J, Burgiel S, Brantley K (2021). Strategic considerations for invasive species managers in the utilization of environmental DNA (eDNA): Steps for incorporating this powerful surveillance tool. Manag Biol Invasions.

[CR52] Mosher BA, Huyvaert KP, Chestnut T (2017). Design- and model-based recommendations for detecting and quantifying an amphibian pathogen in environmental samples. Ecol Evol.

[CR53] Ortmann D (2009) Kammmolch-Monitoring-Krefeld: Populationsökologie einer europaweit bedeutsamen Population des Kammmolches (Triturus cristatus) unter besonderer Berücksichtigung naturschutzrelevanter Fragestellungen

[CR54] Paula DP, Linard B, Crampton-Platt A (2016). Uncovering Trophic Interactions in arthropod predators through DNA shotgun-sequencing of gut contents. PLoS ONE.

[CR55] Pawlowski J, Kelly-Quinn M, Altermatt F (2018). The future of biotic indices in the ecogenomic era: integrating (e)DNA metabarcoding in biological assessment of aquatic ecosystems. Sci Total Environ.

[CR56] Piaggio AJ, Engeman RM, Hopken MW (2014). Detecting an elusive invasive species: a diagnostic PCR to detect Burmese python in Florida waters and an assessment of persistence of environmental DNA. Mol Ecol Resour.

[CR57] Pounds JA, Bustamante MR, Coloma LA (2006). Widespread amphibian extinctions from epidemic disease driven by global warming. Nature.

[CR58] Préau C, Dubech P, Sellier Y (2017). Amphibian response to the non-native fish, lepomis gibbosus: the case of the Pinail Nature Reserve, France. Herpetol Conserv Biol.

[CR59] Riaz M, Kuemmerlen M, Wittwer C (2020). Combining environmental DNA and species distribution modeling to evaluate reintroduction success of a freshwater fish. Ecol Appl.

[CR60] Riaz M, Wittwer C, Nowak C, Cocchiararo B (2018). An environmental DNA assay for the detection of the regionally endangered freshwater fish Alburnoides bipunctatus in Germany. Conserv Genet Resour.

[CR61] Rodgers TW, Xu CCY, Giacalone J (2017). Carrion fly-derived DNA metabarcoding is an effective tool for mammal surveys: Evidence from a known tropical mammal community. Mol Ecol Resour.

[CR62] Rohr JR, Raffel TR (2010). Linking global climate and temperature variability to widespread amphibian declines putatively caused by disease. Proc Natl Acad Sci U S A.

[CR63] Roslin T, Majaneva S (2016). The use of DNA barcodes in food web construction-terrestrial and aquatic ecologists unite!. Genome.

[CR64] Saqib HSA, Liang P, You M, Gurr GM (2021). Molecular gut content analysis indicates the inter- and intra-guild predation patterns of spiders in conventionally managed vegetable fields. Ecol Evol.

[CR65] Sasso T, Lopes CM, Valentini A (2017). Environmental DNA characterization of amphibian communities in the Brazilian Atlantic forest: Potential application for conservation of a rich and threatened fauna. Biol Conserv.

[CR66] Sigsgaard EE, Nielsen IB, Bach SS (2016). Population characteristics of a large whale shark aggregation inferred from seawater environmental DNA. Nat Ecol Evol.

[CR67] Sint D, Kaufmann R, Mayer R, Traugott M (2019). Resolving the predator first paradox: Arthropod predator food webs in pioneer sites of glacier forelands. Mol Ecol.

[CR68] Sodhi NS, Bickford D, Diesmos AC (2008). Measuring the meltdown: drivers of global amphibian extinction and decline. PLoS ONE.

[CR69] Spear MJ, Embke HS, Krysan PJ, Vander Zanden MJ (2020). Application of eDNA as a tool for assessing fish population abundance. Environ DNA.

[CR70] Stevenson DE (2018). Documenting the reliability of species identifications in the North Pacific Observer Program. Fish Res.

[CR71] Stübing S, Hundertmark I (2015). Jahresbericht über die Untersuchungen der Knoblauchkröten-Population im NSG Bingenheimer Ried, Echzell (2014). Büro Für Faun Fachfragen.

[CR72] Sugihara G, May R, Ye H, et al (2012) Detecting causality in complex ecosystems. Science (80- ) 338:496–500. 10.1126/science.1227079 10.1126/science.1227079 22997134

[CR73] Taberlet P, Luikart G, Waits LP (1999). Noninvasive genetic sampling: look before you leap. Trends Ecol Evol.

[CR74] Taberlet P, Coissac E, Pompanon F (2012). Towards next-generation biodiversity assessment using DNA metabarcoding. Mol Ecol.

[CR75] Thalinger B, Deiner K, Harper LR (2021). A validation scale to determine the readiness of environmental DNA assays for routine species monitoring. Environ DNA.

[CR76] Thomsen PF, Sigsgaard EE (2019). Environmental DNA metabarcoding of wild flowers reveals diverse communities of terrestrial arthropods. Ecol Evol.

[CR77] Thomsen PF, Willerslev E (2015). Environmental DNA - An emerging tool in conservation for monitoring past and present biodiversity. Biol Conserv.

[CR78] Thomsen PF, Kielgast J, Iversen LL (2012). Detection of a diverse marine fish fauna using environmental DNA from seawater samples. PLoS ONE.

[CR79] Wilcox TM, Young MK, McKelvey KS (2018). Fine-scale environmental DNA sampling reveals climate-mediated interactions between native and invasive trout species. Ecosphere.

[CR80] Wilcox TM, McKelvey KS, Young MK (2020). Parallel, targeted analysis of environmental samples via high-throughput quantitative PCR. Environ DNA.

[CR81] Wisz MS, Pottier J, Kissling WD (2013). The role of biotic interactions in shaping distributions and realised assemblages of species: Implications for species distribution modelling. Biol Rev.

[CR82] Yamanaka H, Minamoto T (2016). The use of environmental DNA of fishes as an efficient method of determining habitat connectivity. Ecol Indic.

[CR83] Yates MC, Fraser DJ, Derry AM (2019). Meta-analysis supports further refinement of eDNA for monitoring aquatic species-specific abundance in nature. Environ DNA.

[CR84] Ye J, Coulouris G, Zaretskaya I (2012). Primer-BLAST: a tool to design target-specific primers for polymerase chain reaction. BMC Bioinform.

