# Supplementary material for: Using eDNA to understand predator–prey interactions influenced by invasive species
Source: Oecologia. 2023 Aug 18;202(4):757–67. doi: 10.1007/s00442-023-05434-6 (PMC10474997; doi:10.1007/s00442-023-05434-6)
Supplement: Supplementary file 1 — Supplementary file1 (DOCX 55 KB) [file 442_2023_5434_MOESM1_ESM.docx]

**Using eDNA to understand predator-prey interactions influenced by invasive species**

**Maria Riaz^1,2,3*^, Dan Warren^4,5^, Claudia Wittwer^1,2,3^, Berardino Cocchiararo^1,2^, Inga Hundertmark^6^, Tobias Erik Reiners^1,6^_,_ Sven Klimpel^2,3,5^, Markus Pfenninger^2,5,7^, Imran Khaliq^8,9,10^, Carsten Nowak^1,2^**

**^1^**Conservation Genetics Section, Senckenberg Research Institute and Natural History Museum Frankfurt, 63571 Gelnhausen, Germany

^2^LOEWE Centre for Translational Biodiversity Genomics (LOEWE-TBG), Senckenberganlage 25, 60325 Frankfurt am Main, Germany

^3^Faculty of Biological Sciences, Institute for Ecology, Evolution and Diversity, Goethe University, Max-von-Laue-Straße 9, 60438 Frankfurt am Main, Germany

^4^Biodiversity and Biocomplexity Unit, Okinawa Institute of Science and Technology Graduate University, Okinawa, Japan

^5^Senckenberg Biodiversity and Climate Research Centre (BiK-F), Senckenberganlage 25, 60325 Frankfurt am Main, Germany

^6^Hessische Gesellschaft für Ornithologie und Naturschutz (HGON e. V.), Lindenstrasse 5, 61209 Echzell, Germany

^7^Institute for Molecular and Organismic Evolution, Johannes Gutenberg University, Johann-Joachim-Becher-Weg 7, 55128 Mainz, Germany

^8^Department of Education, Punjab, Pakistan

^9^Department of Aquatic Ecology Eawag (Swiss Federal Institute of Aquatic Science and Technology) Überlandstrasse 133, 8600 Dübendorf, Switzerland

^10^Swiss Federal Institute for Forest, Snow and Landscape Research (WSL), Flüelastr. 11, 7260 Davos Dorf, Switzerland

*Author to whom correspondence should be addressed: [hmmmariariaz@yahoo.com](mailto:hmmmariariaz@yahoo.com)

Table 1 Overview of the results obtained by traditional survey across the study sites

| **NO** | **Ponds** | ***Lepomis gibbosus*** | ***Pseudorasbora parva*** | ***Pelobates fuscus*** | ***Triturus cristatus*** | ***Bufo viridis*** | ***Bufo bufo*** | ***Epidalea calamita*** | ***Rana temporaria*** | ***Pelophylax ridibundus*** | ***Pelophylax esculentus*** | ***Hyla arborea*** | ***Lissotriton vulgaris*** | ***Ichthyosaura alpestris*** |
| --- | --- | --- | --- | --- | --- | --- | --- | --- | --- | --- | --- | --- | --- | --- |
| 1 | Steinbruch Bingenheim groß | 1 | 0 | 0 | 0 | 1 | 1 | 0 | 0 | 1 | 1 | 0 | 0 | 0 |
| 2 | Steinbruch Bingenheim klein | 0 | 0 | 0 | 0 | 0 | 1 | 0 | 0 | 0 | 1 | 0 | 0 | 0 |
| 3 | Vermehrungsgewässer | 0 | 1 | 1 | 1 | 1 | 0 | 0 | 0 | 1 | 1 | 1 | 1 | 0 |
| 4 | Grenzstock klein | 0 | 0 | 1 | 1 | 0 | 1 | 0 | 1 | 1 | 1 | 1 | 0 | 0 |
| 5 | Schwelteich klein | 0 | 0 | 1 | 0 | 1 | 1 | 0 | 1 | 1 | 1 | 0 | 1 | 0 |
| 6 | Pfaffensee | 0 | 0 | 0 | 0 | 1 | 0 | 0 | 0 | 1 | 1 | 1 | 1 | 0 |
| 7 | Pfaffensee Kleingewässer 1 | 0 | 0 | 1 | 0 | 1 | 0 | 0 | 0 | 1 | 1 | 0 | 1 | 0 |
| 8 | Pfaffensee Flachgewässer | 0 | 0 | 0 | 0 | 1 | 0 | 1 | 1 | 1 | 1 | 1 | 1 | 0 |
| 9 | Pfaffensee Kleingewässer 2 | 0 | 0 | 1 | 0 | 1 | 0 | 0 | 0 | 1 | 1 | 0 | 1 | 0 |
| 10 | Grubenteich klein | 0 | 0 | 1 | 1 | 0 | 1 | 0 | 0 | 1 | 1 | 1 | 1 | 0 |
| 11 | Biedrichsgraben | 1 | 0 | 1 | 0 | 0 | 1 | 0 | 0 | 1 | 1 | 1 | 1 | 0 |
| 12 | Stockborn groß | 0 | 1 | 0 | 0 | 0 | 1 | 0 | 1 | 1 | 1 | 1 | 1 | 1 |
| 13 | Stockborn klein | 0 | 1 | 1 | 1 | 1 | 1 | 0 | 1 | 1 | 1 | 1 | 1 | 1 |
| 14 | Stockborn I | 0 | 0 | 0 | 0 | 0 | 0 | 0 | 0 | 0 | 0 | 1 | 1 | 1 |
| 15 | Stockborn II | 0 | 0 | 0 | 1 | 0 | 0 | 0 | 0 | 0 | 1 | 1 | 1 | 1 |
| 16 | Stockborn III | 0 | 0 | 1 | 1 | 0 | 1 | 0 | 0 | 1 | 1 | 1 | 1 | 1 |
| 17 | Leidhecken | 0 | 0 | 0 | 1 | 0 | 0 | 0 | 1 | 1 | 1 | 1 | 1 | 0 |
| 18 | Reichelsheim alt | 0 | 1 | 1 | 0 | 0 | 1 | 0 | 1 | 1 | 1 | 1 | 1 | 0 |
| 19 | Reichelsheim neu | 0 | 1 | 1 | 0 | 0 | 1 | 0 | 1 | 1 | 1 | 1 | 1 | 0 |

Overview of the results of traditional monitoring data of amphibian and fish species across the study ponds. Presence sand absence of each species is represented by “1” and “0” respectively

Table 2 Overview of the results obtained by eDNA survey during all sampling months across all sampling sites

| **NO.** | **Site** | **Months** | ***Lepomis gibbosus* Cq value** | ***Lepomis gibbosus* DNA concentration (ng/μl)** | ***Pelobates fuscus* Cq value** | ***Pelobates fuscus* DNA concentration (ng/μl)** | ***Pseudorasbora parva* Cq value** | ***Pseudorasbora parva* DNA concentration (ng/μl)** | ***Triturus cristatus* Cq value** | ***Triturus cristatus* DNA concentration (ng/μl)** |
| --- | --- | --- | --- | --- | --- | --- | --- | --- | --- | --- |
| 1 | Biedrichgraben | 5 | 32.83198166 | 0.009559381 | 0 | 0 | 36.455744 | 1.27944E-05 | 0 | 0 |
| 2 | Biedrichgraben | 6 | 31.63454967 | 0.025365217 | 0 | 0 | 35.09588877 | 0.010555345 | 0 | 0 |
| 3 | Biedrichgraben | 7 | 32.04250569 | 0.019364418 | 0 | 0 | 37.26372782 | 0.003152922 | 0 | 0 |
| 4 | Bingenheimer Ried | 5 | 0 | 0 | 33.61045456 | 0.002086737 | 32.91319889 | 0.001279108 | 0 | 0 |
| 5 | Bingenheimer Ried | 6 | 0 | 0 | 30.83571074 | 0.007709689 | 30.32796923 | 0.155300765 | 37.28788884 | 0.001983459 |
| 6 | Bingenheimer Ried | 7 | 33.6781968 | 0.007192407 | 0 | 0 | 30.35429404 | 0.007192407 | 0 | 0 |
| 7 | Breitwiesen 1 | 5 | 0 | 0 | 0 | 0 | 0 | 0 | 39.22341029 | 7.77778E-05 |
| 8 | Breitwiesen 1 | 6 | 0 | 0 | 0 | 0 | 37.86833333 | 0.001710363 | 39.2885437 | 3.64396E-05 |
| 9 | Breitwiesen 1 | 7 | 0 | 0 | 0 | 0 | 39.87928009 | 0.000717282 | 0 | 0 |
| 10 | Breitwiesen 2 | 5 | 0 | 0 | 0 | 0 | 38.33059071 | 0.001806131 | 37.93149334 | 0.000330656 |
| 11 | Breitwiesen 2 | 6 | 0 | 0 | 0 | 0 | 37.86833333 | 0.002386738 | 39.52471733 | 1.51667E-05 |
| 12 | Breitwiesen 2 | 7 | NA | NA | NA | NA | NA | NA | NA | NA |
| 13 | Breitwiesen 3 | 5 | 0 | 0 | 0 | 0 | 32.88571972 | 0.036911506 | 0 | 0 |
| 14 | Breitwiesen 3 | 6 | 0 | 0 | 0 | 0 | 34.34984951 | 0.01583963 | 0 | 0 |
| 15 | Breitwiesen 3 | 7 | 0 | 0 | 0 | 0 | 32.50692728 | 0.045251348 | 0 | 0 |
| 16 | Breitwiesen 4 | 5 | 0 | 0 | 0 | 0 | 28.90829447 | 0.348958936 | 38.40825653 | 0.000246429 |
| 17 | Breitwiesen 4 | 6 | 0 | 0 | 0 | 0 | 34.18132824 | 0.017716719 | 0 | 0 |
| 18 | Breitwiesen 4 | 7 | NA | NA | NA | NA | NA | NA | NA | NA |
| 19 | Breitwiesen 5 | 5 | 0 | 0 | 0 | 0 | 31.48079533 | 0.085005996 | 0 | 0 |
| 20 | Breitwiesen 5 | 6 | 0 | 0 | 0 | 0 | 31.95663812 | 0.062993715 | 0 | 0 |
| 21 | Breitwiesen 5 | 7 | 0 | 0 | 0 | 0 | 32.95144018 | 0.035929677 | 0 | 0 |
| 22 | Grenzstock groß | 5 | 0 | 0 | 0 | 0 | 0 | 0 | 0 | 0 |
| 23 | Grenzstock groß | 6 | 0 | 0 | 0 | 0 | 0 | 0 | 0 | 0 |
| 24 | Grenzstock groß | 7 | 0 | 0 | 0 | 0 | 37.98835945 | 0.002645738 | 0 | 0 |
| 25 | Grenzstock klein | 5 | 0 | 0 | 31.88122961 | 0.005045909 | 0 | 0 | 32.83567725 | 0.005865273 |
| 26 | Grenzstock klein | 6 | 0 | 0 | 26.82279587 | 0.070971224 | 0 | 0 | 28.36849954 | 0.269657118 |
| 27 | Grenzstock klein | 7 | 0 | 0 | 31.58596929 | 0.009130447 | 37.98835945 | 0.002645738 | 30.12979147 | 0.046114059 |
| 28 | Grubenteich groß | 5 | 0 | 0 | 34.31545893 | 0.001404598 | 0 | 0 | 38.7735761 | 0.000114192 |
| 29 | Grubenteich groß | 6 | 0 | 0 | 35.14837986 | 0.000939956 | 0 | 0 | 33.24571398 | 0.011379875 |
| 30 | Grubenteich groß | 7 | 0 | 0 | 36.5511996 | 0.000161092 | 27.57414267 | 1.191622009 | 33.58760325 | 0.006618884 |
| 31 | Grubenteich klein | 5 | 0 | 0 | 0 | 0 | 0 | 0 | 34.35652669 | 0.002443217 |
| 32 | Grubenteich klein | 6 | 0 | 0 | 30.71365145 | 0.015716937 | 0 | 0 | 35.89307446 | 0.002131372 |
| 33 | Grubenteich klein | 7 | 0 | 0 | 38.06569015 | 0.00010555 | 33.33290185 | 0.032907154 | 36.94024065 | 0.000799946 |
| 34 | Leidhecken | 5 | 0 | 0 | 37.85162608 | 0.000123422 | 36.34051132 | 1.97511E-05 | 33.3343349 | 0.010504809 |
| 35 | Leidhecken | 6 | 0 | 0 | 37.23425929 | 0.000121761 | 38.24511111 | 0.002659547 | 35.56442346 | 0.001715247 |
| 36 | Leidhecken | 7 | 0 | 0 | 0 | 0 | 36.04716407 | 0.006480681 | 35.56375249 | 0.001739732 |
| 37 | Leidhecken blofeld | 5 | 0 | 0 | 36.95709907 | 0.000281982 | 0 | 0 | 38.80057928 | 0.000345205 |
| 38 | Leidhecken blofeld | 6 | 0 | 0 | 36.94045258 | 0.000153432 | 0 | 0 | 39.05470339 | 0.000159901 |
| 39 | Leidhecken blofeld | 7 | 0 | 0 | 36.47036192 | 0.000375609 | 0 | 0 | 37.3225068 | 0.000622996 |
| 40 | Pfaffensee 1 | 5 | 0 | 0 | 31.40536181 | 0.012043022 | 0 | 0 | 0 | 0 |
| 41 | Pfaffensee 1 | 6 | 0 | 0 | 37.99095535 | 0.000107271 | 0 | 0 | 0 | 0 |
| 42 | Pfaffensee 1 | 7 | 0 | 0 | 34.35157903 | 0.00148294 | 0 | 0 | 0 | 0 |
| 43 | Pfaffensee 2 | 5 | 0 | 0 | 0 | 0 | 0 | 0 | 38.4721756 | 0.000254207 |
| 44 | Pfaffensee 2 | 6 | 0 | 0 | 38.16911443 | 0.000105004 | 0 | 0 | 36.77596537 | 0.001056542 |
| 45 | Pfaffensee 2 | 7 | 0 | 0 | 38.05312856 | 0.0001094 | 37.68831889 | 0.004005789 | 0 | 0 |
| 46 | Reichelheim alt | 5 | 0 | 0 | 0 | 0 | 30.9624548 | 0.116389755 | 0 | 0 |
| 47 | Reichelheim alt | 6 | 0 | 0 | 0 | 0 | 31.05873723 | 0.106623436 | 0 | 0 |
| 48 | Reichelheim alt | 7 | 0 | 0 | 0 | 0 | 34.29214181 | 0.019202092 | 0 | 0 |
| 49 | Reichelheim neu | 5 | 34.7735121 | 0.005010623 | 0 | 0 | 31.59490797 | 0.097565396 | 0 | 0 |
| 50 | Reichelheim neu | 6 | 36.42495261 | 0.001277723 | 0 | 0 | 32.88571972 | 0.036911506 | 0 | 0 |
| 51 | Reichelheim neu | 7 | 0 | 0 | 0 | 0 | 34.55199136 | 0.01452632 | 0 | 0 |
| 52 | Rockenberg bahndamn | 5 | 0 | 0 | 0 | 0 | 30.28419219 | 0.157913148 | 0 | 0 |
| 53 | Rockenberg bahndamn | 6 | NA | NA | NA | NA | NA | NA | NA | NA |
| 54 | Rockenberg bahndamn | 7 | 0 | 0 | 0 | 0 | 33.11766434 | 0.034241944 | 0 | 0 |
| 55 | Schwelteich groß | 5 | 33.69677778 | 0.007160507 | 0 | 0 | 31.15695635 | 0.095633232 | 0 | 0 |
| 56 | Schwelteich groß | 6 | 34.59480192 | 0.00300113 | 0 | 0 | 36.48854701 | 0.00496692 | 0 | 0 |
| 57 | Schwelteich groß | 7 | 37.00322596 | 0.000786117 | 0 | 0 | 0 | 0 | 0 | 0 |
| 58 | Schwelteich klein | 5 | 0 | 0 | 32.64532089 | 0.019870935 | 0 | 0 | 0 | 0 |
| 59 | Schwelteich klein | 6 | 0 | 0 | 34.17778609 | 0.001767605 | 0 | 0 | 35.27758408 | 0.002040051 |
| 60 | Schwelteich klein | 7 | 0 | 0 | 0 | 0 | 0 | 0 | 0 | 0 |
| 61 | Steinbruch groß | 5 | 33.35544444 | 0.008956452 | 0 | 0 | 0 | 0 | 0 | 0 |
| 62 | Steinbruch groß | 6 | 29.33251678 | 0.096036311 | 0 | 0 | 0 | 0 | 0 | 0 |
| 63 | Steinbruch groß | 7 | 35.05462265 | 0.002195974 | 0 | 0 | 0 | 0 | 0 | 0 |
| 64 | Steinbruch klein | 5 | 0 | 0 | 0 | 0 | 39.63727951 | 0.000817743 | 0 | 0 |
| 65 | Steinbruch klein | 6 | 0 | 0 | 0 | 0 | 0 | 0 | 39.18449211 | 0.000141967 |
| 66 | Steinbruch klein | 7 | 0 | 0 | 0 | 0 | 0 | 0 | 0 | 0 |
| 67 | Stockborn 1 | 5 | 0 | 0 | 0 | 0 | 0 | 0 | 0 | 0 |
| 68 | Stockborn 1 | 6 | 0 | 0 | 0 | 0 | 0 | 0 | 0 | 0 |
| 69 | Stockborn 1 | 7 | 0 | 0 | 0 | 0 | 0 | 0 | 38.11897278 | 0.000103121 |
| 70 | Stockborn 2 | 5 | 0 | 0 | 0 | 0 | 0 | 0 | 0 | 0 |
| 71 | Stockborn 2 | 6 | 0 | 0 | 0 | 0 | 0 | 0 | 0 | 0 |
| 72 | Stockborn 2 | 7 | 0 | 0 | 0 | 0 | 0 | 0 | 37.54061254 | 0.000130236 |
| 73 | Stockborn 3 | 5 | 0 | 0 | 0 | 0 | 0 | 0 | 0 | 0 |
| 74 | Stockborn 3 | 6 | 0 | 0 | 0 | 0 | 0 | 0 | 0 | 0 |
| 75 | Stockborn 3 | 7 | 0 | 0 | 0 | 0 | 0 | 0 | 0 | 0 |
| 76 | Stockborn 4 | 5 | 0 | 0 | 0 | 0 | 0 | 0 | 0 | 0 |
| 77 | Stockborn 4 | 6 | 0 | 0 | 0 | 0 | 0 | 0 | 0 | 0 |
| 78 | Stockborn 4 | 7 | 0 | 0 | 0 | 0 | 0 | 0 | 0 | 0 |
| 79 | Stockborn groß | 5 | 39.19521459 | 0.000213341 | 35.60077778 | 0.002463678 | 29.44378387 | 0.252929469 | 0 | 0 |
| 80 | Stockborn groß | 6 | 39.371 | 0.000259882 | 36.08160683 | 0.000294112 | 31.48079533 | 0.085005996 | 38.96930218 | 0.000157224 |
| 81 | Stockborn groß | 7 | 38.62963443 | 0.000291036 | 37.48960982 | 0.000185431 | 28.90829447 | 0.348958936 | 38.69585609 | 0.000213438 |
| 82 | Stockborn klein | 5 | 0 | 0 | 0 | 0 | 31.06534746 | 0.11448029 | 35.47483826 | 0.002515977 |
| 83 | Stockborn klein | 6 | 0 | 0 | 38.09076246 | 0.000133114 | 34.18132824 | 0.017716719 | 39.18539333 | 0.000114184 |
| 84 | Stockborn klein | 7 | 0 | 0 | 0 | 0 | 29.45913845 | 0.250416895 | 37.66141849 | 0.000673318 |
| 85 | Teich nord+süd | 5 | 0 | 0 | 0 | 0 | 29.85093875 | 0.037857778 | 39.41392422 | 7.45705E-05 |
| 86 | Teich nord+süd | 6 | 0 | 0 | 0 | 0 | 32.80822415 | 0.038160775 | 0 | 0 |
| 87 | Teich nord+süd | 7 | 0 | 0 | 0 | 0 | 27.8542845 | 1.383757264 | 0 | 0 |
| 88 | Vermehrung | 5 | 0 | 0 | 0 | 0 | 32.60946077 | 0.001472051 | 35.81673601 | 0.000963078 |
| 89 | Vermehrung | 6 | NA | NA | NA | NA | NA | NA | NA | NA |
| 90 | Vermehrung | 7 | 0 | 0 | 0 | 0 | 27.1318851 | 1.349541474 | 0 | 0 |
| 91 | Wisselheim | 5 | 0 | 0 | 0 | 0 | 33.12517124 | 0.03187614 | 0 | 0 |
| 92 | Wisselheim | 6 | 0 | 0 | 0 | 0 | 32.65211741 | 0.041163754 | 0 | 0 |
| 93 | Wisselheim | 7 | 0 | 0 | 0 | 0 | 36.18498145 | 0.006093916 | 0 | 0 |

Overview of the results of two prey species, *Pelobates fuscus* (A1), *Triturus cristatus* (A2) and two predator species, *Lepomis gibbosus* (F1), *Pseudorasbora parva* (F2). The three sampling months, May, June and July are represented as 5, 6 and 7 respectively. Presences based on eDNA detections of each species across each sampling site and across each month are shown by Cq values and DNA concentration in ng/μl while, absences are shown by “0”

Table 3 List of fish and amphibian species used for assay specificity tests

| Common Name | Scientific Name |
| --- | --- |
| Atlantic Salmon | *Salmo salar* |
| Brown Trout | *Salmo trutta* |
| Stone Loach | *Barbatula barbatula* |
| Burbot | *Lota lota* |
| Tench | *Tinca tinca* |
| Roach | *Rutilus rutilus* |
| Perch | *Perca fluviatilis* |
| Ruffe | *Gymnocephalus cernua* |
| Dace | *Leuciscus leuciscus* |
| Rainbow Trout | *Oncorhynchus mykiss* |
| Wels Catfish | *Silurus glanis* |
| Three-spined Stickleback | *Gasterosteus aculeatus* |
| Pike-perch | *Sander lucioperca* |
| Gudgeon | *Gobio gobio* |
| Bream | *Abramis brama* |
| Grayling | *Thymallus thymallus* |
| Brook Lamprey | *Lampetra planeri* |
| Common Carp | *Cyprinus carpio* |
| Pumpkinseed | *Lepomis gibbosus* |
| Bighead Goby | *Ponticola kessleri* |
| Round Goby | *Neogobius melanostomus* |
| Asp | *Aspius aspius* |
| Western Tubenose Goby | *Proterorhinus semilunaris* |
| White-finned Gudgeon | *Gobio albipinnatus* |
| Bleak | *Alburnus alburnus* |
| Ide | *Leuciscus idus* |
| European mudminnow | *Umbra krameri* |
| Allis Shad | *Alosa alosa* |
| Weatherfish | *Misgurnus fossilis* |
| Nase | *Chondrostoma nasus* |
| Northern Pike | *Esox lucius* |
| European Eel | *Anguilla anguilla* |
| Barbel | *Barbus barbus* |
| Silver Bream  Common Toad  European Green Toad  Marsh Frog  Smooth Newt  European Tree Frog  Common Frog  Alpine Newt | *Blicca bjoerkna*  *Bufo Bufo*  *Bufotes viridis*  *Pelophylax ridibundus*  *Lissotriton vulgaris*  *Hyla arborea*  *Rana temporaria*  *Ichthyosaura alpestris* |

List of the tested cross species including fish and amphibian species for each TaqMan based species specific assay. Both Latin and common names were given for each cross species

**Table 4** Values of the standard curve parameters obtained for each assay

Values of the standard curve parameters of r^2^_,_ slope of the curve, PCR efficiency, Limit of quantification (LOQ) and Limit of detection (LOD) obtained for each TaqMan based species specific assay

| Standard curve values | *Pseudorasbora parva* | *Lepomis gibbosus* | *Triturus cristatus* | *Pelobates fuscus* |
| --- | --- | --- | --- | --- |
| r^2^ | 0.99 | 0.99 | 0.99 | 0.97 |
| slope | -3.5175 | -3.3618 | -3.3434 | -3.8297 |
| PCR efficiency | 93% | 98% | 99% | 82% |
| Limit of quantification (LOQ) | 38.24 Cq | 36.73Cq | 37.13Cq | 36.33Cq |
| Limit of detection (LOD) | 40.54 Cq | 39.93Cq | 39.76Cq | 38.86Cq |

**Table 5** Sequences obtained from eDNA for Lepomis gibbosus and Pseudorasbora parva

This dataset gives the alignment of *Lepomis gibbosus* and *Pseudorasbora parva* sequences extracted from water samples which are sampled from different ponds during May-July 2018. The a and b represent the different PCR replicates from which the sequences were obtained using species-specific primers with M13 tags on ABI 3730xl Sanger sequencer (Thermo Fisher). This data was assembled using SeqScanner, from ThermoFisher Scientific.

*Lepomis gibbosus*_Steinbruch_gross_July_a

Sequence number: BankIt2706213 Seq1

Accession number: OR053009

TCCACATCGGTCGAGGACTATATTATGGCTCTTACCTTTACAAAGAAACATGAAACATCGGAGTCG

*Lepomis gibbosus*_Steinbruch_gross_July_b

Sequence number: BankIt2706213 Seq2

Accession number: OR053010

TCCACATCGGTCGAGGACTATATTATGGCTCTTACCTTTACAAAGAAACATGAAACATCGGAGTCG

*Lepomis gibbosus*_Steinbruch_gross_July_c

Sequence number: BankIt2706213 Seq3

Accession number: OR053011

TCCACATCGGTCGAGGACTATATTATGGCTCTTACCTTTACAAAGAAACATGAAACATCGGAGTCG

*Lepomis gibbosus*_Steinbruch_gross_July_d

Sequence number: BankIt2706213 Seq4

Accession number: OR053012

TCCACATCGGTCGAGGACTATATTATGGCTCTTACCTTTACAAAGAAACATGAAACATCGGAGTCG

*Lepomis gibbosus*_Steinbruch_gross_June_a

Sequence number: BankIt2706213 Seq5

Accession number: OR053013

TCCACATCGGTCGAGGACTATATTATGGCTCTTACCTTTACAAAGAAACATGAAACATCGGAGTCG

*Lepomis gibbosus*_Steinbruch_gross_June_b

Sequence number: BankIt2706213 Seq6

Accession number: OR053014

TCCACATCGGTCGAGGACTATATTATGGCTCTTACCTTTACAAAGAAACATGAAACATCGGAGTCG

*Lepomis gibbosus*_Steinbruch_gross_June_c

Sequence number: BankIt2706213 Seq7

Accession number: OR053015

TCCACATCGGTCGAGGACTATATTATGGCTCTTACCTTTACAAAGAAACATGAAACATCGGAGTCG

*Lepomis gibbosus*_Steinbruch_gross_June_d

Sequence number: BankIt2706213 Seq8

Accession number: OR053016

TCCACATCGGTCGAGGACTATATTATGGCTCTTACCTTTACAAAGAAACATGAAACATCGGAGTCG

*Lepomis gibbosus*_Schwelteich_gross_June_a

Sequence number: BankIt2706213 Seq9

Accession number: OR053017

TCCACATCGGTCGAGGACTATATTATGGCTCTTACCTTTACAAAGAAACATGAAACATCGGAGTCG

*Lepomis gibbosus*_Schwelteich_gross_June_b

Sequence number: BankIt2706213 Seq10

Accession number: OR053018

TCCACATCGGTCGAGGACTATATTATGGCTCTTACCTTTACAAAGAAACATGAAACATCGGAGTCG

*Lepomis gibbosus*_Schwelteich_gross_June_c

Sequence number: BankIt2706213 Seq11

Accession number: OR053019

TCCACATCGGTCGAGGACTATATTATGGCTCTTACCTTTACAAAGAAACATGAAACATCGGAGTCG

*Lepomis gibbosus*_Schwelteich_gross_June_d

Sequence number: BankIt2706213 Seq12

Accession number: OR053020

TCCACATCGGTCGAGGACTATATTATGGCTCTTACCTTTACAAAGAAACATGAAACATCGGAGTCG

*Lepomis gibbosus*_Biedrichgraben_May_a

Sequence number: BankIt2706213 Seq13

Accession number: OR053021

TCCACATCGGTCGAGGACTATATTATGGCTCTTACCTTTACAAAGAAACATGAAACATCGGAGTCG

*Lepomis gibbosus*_Biedrichgraben_May_b

Sequence number: BankIt2706213 Seq14

Accession number: OR053022

TCCACATCGGTCGAGGACTATATTATGGCTCTTACCTTTACAAAGAAACATGAAACATCGGAGTCG

*Lepomis gibbosus*_Biedrichgraben_May_c

Sequence number: BankIt2706213 Seq15

Accession number: OR053023

TCCACATCGGTCGAGGACTATATTATGGCTCTTACCTTTACAAAGAAACATGAAACATCGGAGTCG

*Lepomis gibbosus*_Biedrichgraben_May_d

Sequence number: BankIt2706213 Seq16

Accession number: OR053024

TCCACATCGGTCGAGGACTATATTATGGCTCTTACCTTTACAAAGAAACATGAAACATCGGAGTCG

*Lepomis gibbosus*_tissue_a

Sequence number: BankIt2706213 Seq17

Accession number: OR053025

TCCACATCGGTCGAGGACTATATTATGGCTCTTACCTTTACAAAGAAACATGAAACATCGGAGTCG

*Lepomis gibbosus*_tissue_b

Sequence number: BankIt2706213 Seq18

Accession number: OR053026

TCCACATCGGTCGAGGACTATATTATGGCTCTTACCTTTACAAAGAAACATGAAACATCGGAGTCG

*Pseudorasbora parva*_Teich_Nord_May_a

Sequence number: BankIt2706213 Seq19

Accession number: OR053027

AACAGGACTATTCTTGGCCATACACTACACCTCTGACATCTCAACTGCATTTTCATCGGTGGCCCACATC

*Pseudorasbora parva*_Teich_Nord_May_b

Sequence number: BankIt2706213 Seq20

Accession number: OR053028

AACAGGACTATTCTTGGCCATACACTACACCTCTGACATCTCAACTGCATTTTCATCGGTGGCCCACATC

*Pseudorasbora parva*_Teich_Nord_May_c

Sequence number: BankIt2706213 Seq21

Accession number: OR053029

AACAGGACTATTCTTGGCCATACACTACACCTCTGACATCTCAACTGCATTTTCATCGGTGGCCCACATC

*Pseudorasbora parva*_Teich_Nord_May_d

Sequence number: BankIt2706213 Seq22

Accession number: OR053030

AACAGGACTATTCTTGGCCATACACTACACCTCTGACATCTCAACTGCATTTTCATCGGTGGCCCACATC

*Pseudorasbora parva*_Biedrichgraben_May_a

Sequence number: BankIt2706213 Seq23

Accession number: OR053031

AACAGGACTATTCTTGGCCATACACTACACCTCTGACATCTCAACTGCATTTTCATCGGTGGCCCACATC

*Pseudorasbora parva*_Biedrichgraben_May_b

Sequence number: BankIt2706213 Seq24

Accession number: OR053032

AACAGGACTATTCTTGGCCATACACTACACCTCTGACATCTCAACTGCATTTTCATCGGTGGCCCACATC

*Pseudorasbora parva*_Biedrichgraben_May_c

Sequence number: BankIt2706213 Seq25

Accession number: OR053033

AACAGGACTATTCTTGGCCATACACTACACCTCTGACATCTCAACTGCATTTTCATCGGTGGCCCACATC

*Pseudorasbora parva*_Biedrichgraben_May_d

Sequence number: BankIt2706213 Seq26

Accession number: OR053034

AACAGGACTATTCTTGGCCATACACTACACCTCTGACATCTCAACTGCATTTTCATCGGTGGCCCACATC

*Pseudorasbora parva*_Schwelteich_gross_June_a

Sequence number: BankIt2706213 Seq27

Accession number: OR053035

AACAGGACTATTCTTGGCCATACACTACACCTCTGACATCTCAACTGCATTTTCATCGGTGGCCCACATC

*Pseudorasbora parva*_Schwelteich_gross_June_b

Sequence number: BankIt2706213 Seq28

Accession number: OR053036

AACAGGACTATTCTTGGCCATACACTACACCTCTGACATCTCAACTGCATTTTCATCGGTGGCCCACATC

*Pseudorasbora parva*_Schwelteich_gross_June_c

Sequence number: BankIt2706213 Seq29

Accession number: OR053037

AACAGGACTATTCTTGGCCATACACTACACCTCTGACATCTCAACTGCATTTTCATCGGTGGCCCACATC

*Pseudorasbora parva*_Schwelteich_gross_June_d

Sequence number: BankIt2706213 Seq30

Accession number: OR053038

AACAGGACTATTCTTGGCCATACACTACACCTCTGACATCTCAACTGCATTTTCATCGGTGGCCCACATC

*Pseudorasbora parva*_Stockborn_gross_May_a

Sequence number: BankIt2706213 Seq31

Accession number: OR053039

AACAGGACTATTCTTGGCCATACACTACACCTCTGACATCTCAACTGCATTTTCATCGGTGGCCCACATC

*Pseudorasbora parva*_Stockborn_gross_May_b

Sequence number: BankIt2706213 Seq32

Accession number: OR053040

AACAGGACTATTCTTGGCCATACACTACACCTCTGACATCTCAACTGCATTTTCATCGGTGGCCCACATC

*Pseudorasbora parva*_Stockborn_gross_May_c

Sequence number: BankIt2706213 Seq33

Accession number: OR053041

AACAGGACTATTCTTGGCCATACACTACACCTCTGACATCTCAACTGCATTTTCATCGGTGGCCCACATC

*Pseudorasbora parva*_Stockborn_gross_May_d

Sequence number: BankIt2706213 Seq34

Accession number: OR053042

AACAGGACTATTCTTGGCCATACACTACACCTCTGACATCTCAACTGCATTTTCATCGGTGGCCCACATC

*Pseudorasbora parva*_tissue_a

Sequence number: BankIt2706213 Seq35

Accession number: OR053043

AACAGGACTATTCTTGGCCATACACTACACCTCTGACATCTCAACTGCATTTTCATCGGTGGCCCACATC

*Pseudorasbora parva*_tissue_b

Sequence number: BankIt2706213 Seq36

Accession number: OR053044

AACAGGACTATTCTTGGCCATACACTACACCTCTGACATCTCAACTGCATTTTCATCGGTGGCCCACATC

**Table 6** List of the study ponds with Global Positioning System (GPS) information

| NO. | Site name | Y co-ordinate | X co-ordinate |
| --- | --- | --- | --- |
| 1 | Vermehrung | 8.876155 | 50.370318 |
| 2 | Pfaffensee 1 | 8.866179 | 50.375054 |
| 3 | Pfaffensee 2 | 8.866278 | 50.375694 |
| 4 | Teich nord+süd | 8.881141 | 50.374431 |
| 5 | Bingenheimer Ried | 8.881667 | 50.368861 |
| 6 | Grubenteich groß | 8.856571 | 50.375545 |
| 7 | Grubenteich klein | 8.858222 | 50.376972 |
| 8 | Grenzstock groß | 8.840583 | 50.381417 |
| 9 | Grenzstock klein | 8.840428 | 50.382987 |
| 10 | Schwelteich groß | 8.855333 | 50.398111 |
| 11 | Schwelteich klein | 8.857282 | 50.397900 |
| 12 | Biedrichgraben | 8.870861 | 50.390889 |
| 13 | Stockborn 1 | 8.898056 | 50.380583 |
| 14 | Stockborn 2 | 8.897778 | 50.380694 |
| 15 | Stockborn 3 | 8.897778 | 50.380917 |
| 16 | Stockborn 4 | 8.897610 | 50.381175 |
| 17 | Stockborn klein | 8.896861 | 50.381194 |
| 18 | Stockborn groß | 8.895805 | 50.380611 |
| 19 | Leidhecken | 8.890667 | 50.350028 |
| 20 | Leidhecken blofeld | 8.896056 | 50.355639 |
| 21 | Reichelheim neu | 8.887966 | 50.347648 |
| 22 | Reichelheim alt | 8.888111 | 50.348997 |
| 23 | Steinbruch groß | 8.901017 | 50.365123 |
| 24 | Steinbruch klein | 8.901389 | 50.367728 |
| 25 | Wisselheim | 8.751583 | 50.388806 |
| 26 | Rockenberg bahndamn | 8.732278 | 50.422167 |
| 27 | Breitwiesen 1 | 8.738361 | 50.403806 |
| 28 | Breitwiesen 2 | 8.738778 | 50.404694 |
| 29 | Breitwiesen 3 | 8.738805 | 50.405111 |
| 30 | Breitwiesen 4 | 8.741806 | 50.406250 |
| 31 | Breitwiesen 5 | 8.737722 | 50.404639 |

Global Positioning System (GPS, Datum: WGS84) information of all the sampled ponds. These sampling sites are located in seven locally important nature protection areas of “Auenverbund Wetterau” which is a landscape protection area within the European Natura 2000 network of protected areas (AGAR and Fena 2010)
